# Genetic and Molecular Differences in Prostate Carcinogenesis between African American and Caucasian American Men

**DOI:** 10.3390/ijms140815510

**Published:** 2013-07-25

**Authors:** James Farrell, Gyorgy Petrovics, David G. McLeod, Shiv Srivastava

**Affiliations:** 1 Center for Prostate Disease Research, Department of Surgery, Uniformed Services University of the Health Sciences, Bethesda, Maryland, 1530 E. Jefferson St., Rockville, MD 20852, USA; E-Mails: james.s.farrell@gmail.com (J.F.); gpetrovics@cpdr.org (G.P.); dgmcleod@verizon.net (D.G.M.); 2 Urology Service, Walter Reed National Military Medical Center, Bethesda, MD 20889, USA

**Keywords:** prostate cancer, ERG, racial differences, androgen receptor, CAG repeats, GWAS, review

## Abstract

Prostate cancer is the most common non-skin cancer and the second leading cause of cancer-related death for men in the United States. Prostate cancer incidence and associated mortality are highest in African American men in comparison to other races. The observed differences in incidence and disease aggressiveness at presentation support a potential role for different pathways of prostate carcinogenesis between African American and Caucasian men. This review focuses on some of the recent molecular biology discoveries, which have been investigated in prostate carcinogenesis and their likely contribution to the known discrepancies across race and ethnicity. Key discussion points include the androgen receptor gene structure and function, genome-wide association studies and epigenetics. The new observations of the ethnic differences of the ERG oncogene, the most common prostate cancer gene, are providing new insights into ERG based stratification of prostate cancers in the context of ethnically diverse patient populations. This rapidly advancing knowledge has the likely potential to benefit clinical practice. Current and future work will improve the ability to sub-type prostate cancers by molecular alterations and lead to targeted therapy against this common malignancy.

## 1. Introduction

In 2013 an estimated 238,590 men will be diagnosed with carcinoma of the prostate (CaP) and an estimated 29,720 men will die from the disease [1]. This malignancy is the second leading cause of cancer-related death in men in the United States. In addition, African American (AA) men have the highest incidence and mortality from CaP compared with other races [1]. The racial disparity exists from presentation and diagnosis through treatment, survival, and quality of life [2]. Researchers have suggested that socio-economic status (SES) contributes significantly to these disparities including CaP-specific mortality [3]. As well, there is evidence that reduced access to care is associated with poor CaP outcomes, which is more prevalent among AA men than Caucasian American (CA) men [4]. While ethnic differences in CaP incidence have been noted across the world, this review focuses on differences between AA and CA men.

There are populations in which AA men have similar outcomes to CA men. Sridhar and colleagues [5] published a meta-analysis in which they concluded that when SES is accounted for, there are no differences in the overall and CaP-specific survival between AA and CA men. Similarly, the military and veteran populations (systems of equal access and screening) do not observe differences in survival across race [6], and differences in pathologic stage at diagnosis narrowed by the early 2000s in a veterans’ cohort [7]. Of note, both of these studies showed that AA men were more likely to have higher Gleason scores and PSA levels than CA men [6,7].

While socio-economic factors contribute to CaP outcomes, they do not seem to account for all variables associated with the diagnosis and disease risk. Several studies support that AA men have a higher incidence of CaP compared to CA men [1,8,9]. Studies also show that AA men have a significantly higher PSA at diagnosis, higher grade disease on biopsy, greater tumor volume for each stage, and a shorter PSA doubling time before radical prostatectomy [10–12]. Biological differences between prostate cancers from CA and AA men have been noted in the tumor microenvironment with regard to stress and inflammatory responses [13]. Although controversy remains over the role of biological differences, observed differences in incidence and disease aggressiveness at presentation suggest a potential role for different pathways of prostate carcinogenesis between AA and CA men. This review focuses on genetic, molecular, and environmental influences, which have been investigated in CaP that contribute to differences between AA and CA men regarding prostate carcinogenesis.

## 2. Androgen and Estrogen Levels

Androgen biosynthesis and androgen receptor (AR) mediated signaling are targets in CaP treatment, and the importance of androgen deprivation therapy is well established [14,15]. However, the role androgens play in CaP risk and development is less well defined. Androgens are necessary for the growth and development of the prostate from fetal life through adulthood. They stimulate cell proliferation and differentiation, and researchers hypothesize that multiple activation pathways are involved in disordered cell growth [16]. This hypothesis was initially supported by findings that a small group of men with CaP had significantly increased testosterone compared with controls [17]. The biological role of sex hormones has been strengthened by animal models, which showed that testosterone caused CaP in rat models; and when combined with estrogen, cancer developed more quickly [18,19]. Further animal studies have shown that both testosterone and estrogen are important for carcinogenesis [20]. Molecular research also supports a role for estrogen and estrogen receptors (ER) in CaP, particularly in concert with inflammation and reactive oxygen species [20,21].

Observational studies carried out in humans assessed whether circulating sex steroid hormone levels, primarily testosterone, affect the risk of CaP and the results are contradictory [20,22–26]. Perhaps the best evidence to support the androgen hypothesis in humans are the results of trials with alpha-5 reductase inhibitors. Dutasteride and finasteride inhibit the conversion of testosterone to dihydrotestosterone (DHT), which is an integral growth hormone for the prostate. In two well known studies, these drugs reduced the prevalence of CaP by 23%–25% over 4–7 years of follow up [27,28]. In addition, these studies showed the presence of more aggressive disease in a subset of men treated with finasteride, suggesting the selection of more aggressive CaP cells under attenuated DHT signaling in the prostate [28].

There are data to support that circulating and intra-prostatic levels of estrogen increase as men age, and the testosterone/estrogen ratio decreases with age; aromatase expression may be involved in this process [21,29]. Similar to the CaP prevention trials, investigators have studied whether reducing that action of estrogen with a selective estrogen receptor modulator (Toremifene) lowers CaP risk [30]. Patients with prostatic intra-epithelial neoplasia (PIN) who were on Toremifene for 1 year had a reduced risk of CaP incidence compared to placebo of 9.1% *vs.* 17.4% respectively [30]. However, when a trial with Toremifene was extended to 3 years, no significant risk reduction was observed [31].

Researchers have evaluated whether sex steroid exposure could contribute to the racial disparity of CaP incidence, and this is a plausible hypothesis. AA men have higher serum and free testosterone levels compared with CA men in a younger male cohort [32]. Estradiol and sex hormone binding globulin (SHBG) have also been found to be higher in AA men [33–35]. The effect of this observation is strengthened by the correlation of increasing estrogen levels with age, potential estrogen effects on carcinogenesis, and the high incidence of CaP in AA men [21]. However, many studies on sex hormone differences across race tend to focus on younger men who have not been followed longitudinally for prostate disease. Data regarding serum testosterone later in life do show that AA and CA men have similar levels at time of prostate biopsy and in their prostate biopsy tissue [36,37]. While the belief that androgens and (more recently) estrogens are key to CaP remains valid, their role in carcinogenesis is not clear [20]. At this time there is no clear, consistent evidence to support the hypothesis that either circulating or intra-tumoral sex hormone levels have a major causal effect on racial variations of CaP.

## 3. Androgen Receptor Gene Structure and Function

### 3.1. Androgen Receptor (AR) Gene Structure

The AR is an essential protein for normal growth and development of male organs, including the prostate. Extensive research suggests the AR is integral in the development and progression of CaP. Structure and function studies on the AR have been a focal point for researchers over the past two decades and racial variations are being elucidated. One area is structural polymorphisms and how androgen activity is assessed along with carcinogenesis and variations across ethnicities.

The AR gene is located on chromosome Xq11-12 and consists of 8 exons. The first exon has a domain, which is thought to control transcriptional activation. Exon 1 became an area of interest after researchers demonstrated that an increased number of CAG repeats (> than 40) in exon 1 are associated with Kennedy’s disease, a mild to moderate androgen insensitivity syndrome associated with spinobulbar muscle atrophy [38]. The variation of CAG repeats in the DNA of normal men is between 11 and 31 repeats [39]. Subsequent studies demonstrated that androgen-dependent transcriptional activity is inversely related to the length of exon 1 based on the number of CAG repeats [38,40,41]. Men with few repeats tend to have a more active AR, and studies have shown that AA men tend to have fewer CAG repeats than CA men [42–44]. Multiple studies have quantified that AA men are significantly more likely to have fewer than 20 CAG repeats compared with CA men [42,45].

The risk of aggressive CaP has been associated with shorter CAG repeats in a relatively large cohort of younger men [46]. Additionally, Ingles and colleagues [47] observed that the risk of developing advanced CaP significantly increased in men with fewer than 20 repeats. These studies (1) supported the theory that CaP is, at least in part, driven by androgen receptor activity, and (2) suggested a genetic mechanism to account for the increased incidence of CaP in AA men [48]. However, several studies found no association between the number of AR CAG repeats and CaP [49–52]. In an editorial by Giovannucci in 2002, he noted that the studies without an association were conducted during the PSA era which enabled detection of less aggressive cancer phenotypes [48]. He reiterated that the strongest studies in support of the CAG repeat theory were prior to wide-spread PSA screening, which were based on symptoms or rectal exam findings and not affected by the lead-time bias of PSA. He also noted that these cancers occurred before age 60. Subsequently he speculated on a “two pool” model; *i.e.*, reduced CAG repeats enable a state of increased androgenicity and androgen-driven CaP in younger men. Later in life, CaP may involve androgens, but may be more driven by accumulated genetic, inflammatory, and environmental factors [48]. However, this “two pool” hypothesis has not been fully elucidated, and while CAG repeats may have a role in CaP, their detection in the PSA era has rendered them less relevant.

### 3.2. Androgen Receptor Function

The gene structure of the AR is obviously important; however, its function is more important. The AR has genomic and non-genomic functions [16,53]. While AR function has been difficult to quantify, one salient observation is the fact that AR protein expression has been evaluated in men with CaP and differences across race have been noted. Gaston and colleagues [54] assessed AR protein expression in malignant and benign prostate tissue between black and white patients. AR immunohistochemistry (IHC) was performed, visual scoring and mean optical density were calculated, and significant differences were noted [54]. The authors discerned that AA men were 27% more likely to stain positive for the AR, and in CaP patients, nuclear expression of the AR was 81% greater in AA men compared with CA men. There was a trend noted in the benign tissue as well, though not as strongly. These observations suggest that the genomic function of the AR may be higher in AA men. The authors noted that if racial differences are responsible, then the mechanism is complex. The investigators postulated that AR protein levels may be increased in part by stabilization from androgens or enhanced mRNA expression [54].

In addition to AR protein expression, common AR transcription targets have been analyzed in benign and malignant cells as a surrogate of AR function [55]. In this study, malignant prostate cells expressed significantly less PSA mRNA compared to benign cells, and lower PSA in CaP cells was associated with increased biochemical recurrence and less time to recurrence [55]. Dobi and colleagues [56] expanded this concept to develop an index of AR function. Also discerned were androgen inducible genes with rapid and strong expression. This factor, plus cumulative gene expression were applied to clinicopathologic parameters with the intent to predict outcomes based on AR pathway dysfunction [56]. While the current efforts are to direct treatment, the index may also be valuable for CaP risk stratification. The impact of race has not been evaluated by this method, and follow up studies regarding protein expression in CaP tissue are ongoing. Other investigators recently published that prostate biopsies from black men express significantly higher levels of CaP biomarkers than those of white men, one of which was the AR [57]. Currently, there is not much known about the functional differences of the AR across race. Recent studies highlight the role of androgen biosynthesis and AR expression in CaP and castration resistant CaP [14,15]. This pathway and tumor biology are beginning to be evaluated for differences between AA and CA men (see below). Future research efforts in these areas are needed [13].

## 4. Genetic Variation

Inherited genes are thought to have a strong influence over the development of CaP [58,59]. Zeegers *et al.* [60] reviewed over 30 epidemiologic studies on CaP and showed that men with a first-degree family member with CaP are 2.5 times more likely to develop CaP. The risk was greater for those with an affected brother (3.37 times) compared to the risk from having a father with CaP (2.17 times). Additionally, risk increased by a factor of 5 if two or more first-degree relatives were affected [60]. Genetic linkage studies show that family history is a well-established marker of risk for CaP.

CaP is the most common solid organ malignancy in men, and 1 in 6 men will be diagnosed with the disease in their lifetime [1]. In addition, most men diagnosed with CaP do not have a family history. For this reason, many researchers doubt that CaP develops from a few inherited alleles with strong penetrance [61,62]. The disease most likely develops from the interaction of several alleles with variable penetrance in combination with other environmental or inflammatory influences [61,63]. To help elucidate common alleles across unrelated populations, genome-wide association studies (GWAS) are being conducted. Studies have identified more than 40 inherited alleles associated with CaP and differences have been noted between AA and CA men [58,61].

Through GWAS many single nucleotide polymorphisms (SNPs) have been identified on several chromosomes and may be associated with CaP risk [58]. Due to the incident difference of SNPs and CaP across ethnicities, recent research has attempted to attribute CaP risk with common SNPs. While loci concerning for CaP risk have been found on chromosomes 7, 10, 11, 19, and others [64,65]; the most compelling risk alleles for CaP are on a segment of chromosome 8q24. Early studies placed the attributable risk of a variant allele on 8q24 at 8% for men of European ancestry and the risk of CaP for AA men with this variant at 16% [66]. The authors hypothesized that this difference may contribute to the incident difference between the ethnicities [66].

Freedman and colleagues [67] used whole-genome admixture mapping to conclude independently that a 3.8 million base pair (Mb) region on chromosome 8q24 is significantly associated with risk for CaP. They further observed that the increased risk of the 8q24 allele in African ancestry is age related and the risk attenuates after age 72 [67]. Based on their results, they speculated that if AA men had two copies of the 8q24 alleles of European Americans, there would be a 49% decrease in the incidence of CaP in AA men. In their study, a 3.8 Mb interval had a single admixture peak that contained nine genes [67]. A follow-up study selected SNPs at 8q24 from the International HapMap Project [68]. The authors identified seven SNPs that were able to account for the signal peak observed by Freedman *et al.* in their cohort of men from African descent [68]. Of those seven, SNP variants rs16901979, rs7000448, and rs6983267 at 8q24 were found to have the strongest associations and confer significant risk for CaP in men of African ancestry [68]. However, none of the SNP variants aligned with a known gene [68].

With several SNPs having significantly increased prevalence among men who develop CaP, a logical step was to evaluate the level of CaP risk that these SNPs confer. Zheng and colleagues [69] showed that men with a family history of CaP along with SNPs in several chromosomal regions had an odds ratio for CaP of 9.46 compared to men without any of the factors tested. However, when SNPs were included in risk models along with age and family history, the positive predictive value did not equal the predictive value of PSA [70]. The authors looked at receiver-operator curves and found that the area under the curve (AUC) increased from 0.58 for model 1 (age), to 0.61 in model 2 (age + family history), and to 0.65 for model 3 (age + family history + 11 risk SNPs) [70]. They compared their results against the performance of PSA alone from the Prostate Cancer Prevention Trial group, where the AUC was 0.68 [70,71].

Despite only a modest predictive improvement beyond age and family history, SNP variants are still attractive for many reasons. Significant differences have been noted in men who develop CaP and SNP variants have been accentuated by ethnicity. Since genetic material does not change throughout one’s life, a SNP risk panel could be created, measured once, and help assess a patient’s risk for CaP. Hughes and colleagues [72] recently applied this concept. They followed a cohort of mostly AA men at risk for CaP, evaluated 6 SNPs associated with CaP, and compared predictive accuracy with PSA. Among AA men, rs6983561 SNP correlated significantly with early-onset CaP. In combination with PSA, the predictive accuracy increased from 0.57 (PSA only) to 0.75 [72].

However, there are two separate issues with the use of SNP variants and their application in CaP. First, these variants are mostly in intronic and intergenic regions of DNA, which makes links to gene expression more uncertain [58]. In addition, little is known about these varients’ relationship to CaP aggressiveness. The 8q24 region is near *C-MYC*, a well-known proto-oncogene, and potential associations have been studied extensively [73,74]. Unfortunately, a link between SNPs and *C-MYC* gene expression has been difficult to prove. A CaP risk variant (rs6983267) within 8q24 was identified in a prostate enhancer with similar expression to *C-MYC* during phases of prostate organogenesis [75]. The authors speculated that rs6983267 may play a role in tissue growth prior to carcinogenesis. Also, amplification of the *C-MYC* oncogene has been associated with CaP recurrence and metastasis, but in that study *C-MYC* related protein expression was not [76].

Six SNPs at 8q24 were examined for increased miRNA expression in prostatectomy tissue, and no significant association was found [73]. A recent study on potential biomarkers in AA men with metastatic CaP suggested that gain of the miR151 gene on 8q24 was more common in patients with a poor outcome, and gain of this miRNA has been associated with enhanced cell motility and invasion in hepatocellular carcinoma [77,78]. In a thorough review, Huppi *et al.* [79] addressed the complexity between 8q24 and *C-MYC*. They noted that a clear mechanism for variants at 8q24 that utilize *C-MYC* expression to cause carcinogenesis or progression has not yet been established [79].

Similar types of results have been noted for SNPs associated with CaP risk on other chromosomes. A SNP variant on chromosome 10, rs10993994, is associated with the decreased expression of the prostatic cell-growth regulator gene *MSMB* [64,80] and it has potential as a urine-based biomarker [81]. On chromosome 17, rs7210100 is in an intron of a zinc-finger gene associated with protein transcription. However, its prevalence in African ancestry is only 4%–7% although its prevalence is <1% in other ethnicities [82].

Allelic variations in genes of the *CYP3A* family, which influence androgen metabolism, have been evaluated and reviewed [83]. There are some race specific differences to support transcripts of *CYP17* and *CYP3A4* as CaP candidate genes, but consistent evidence is lacking [84]. Polymorphisms of *CYP17* have been associated with improved CaP survival in CA men, but more recently the rs743572 SNP of *CYP17* was associated with an increased risk of CaP in AA men [85,86].

Some studies have reported linking GWAS risk SNPs and CaP aggressiveness. The 8q24 risk alleles were tested against clinical variables in a case series of AA men [87]. Within 8q24, the Broad11934905 A risk allele is exclusive to African ethnicity. It was associated with non-organ-confined CaP at time of radical prostatectomy (RP) and a trend toward earlier biochemical recurrence [87]. Two SNPs (rs16901979 on 8q24 and rs7931342 on 11q13) have been significantly associated with CaP-specific mortality and the former with all cause mortality [88]; SNPs on 19q13 near *KlK3* have been associated with serum PSA levels in AA men [65]. Overall, studies like these are smaller and remain to be validated. While GWAS on SNPs hold enormous potential to scan populations for risk alleles, they have not demonstrated the consistent ability to detect markers of aggressive disease. Unfortunately, this approach may not enhance our current ability to screen for at-risk populations [62,69].

## 5. Epigenetic Changes

Substantial evidence exists to support a role for epigenetic changes in the development of CaP [89,90] and some studies have examined differences across ethnicity. Excitement surrounds epigenetic targets as therapeutics for cancer because the cancerous changes are potentially reversible, and the DNA remains intact [91]. Atypical hypermethylation of promoter CpG islands has been shown to increase with age in normal prostate tissue, and in the same study, CaP tissue showed significantly higher methylation levels of several genes including glutathione *S*-transferase-pi gene (*GSTP1*) [92]. Glutathione *S*-transferase is a protein that functions in drug metabolism and is a free radical scavenger. Hypermethylation of *GSTP1* is rare in normal and hyperplastic prostatic epithelium; however, it has been found in 70% of high-grade PIN and in greater than 90% of CaP tissue [93]. Kwabi-Addo and colleagues [94] followed up their 2007 study by comparing DNA methylation status between AA and CA men in matched samples of prostate cancer tissue. They evaluated genes with regulatory roles in prostate disease, including *GTSP1*, the *AR*, *SPARC*, *TIMP3*, and *NKX2-5*. For each gene except *GTSP1*, the authors observed significantly greater methylation in CaP tissue of AA men compared to CA men. *NKX2-5* and *TIMP3* were significantly more likely to be methylated in normal prostate tissue of AA men, and an association with older age in AA men was noted for methylated *NKX2-5* [94]. These findings also support a role for methylation by environmental and inflammatory processes which accumulate over years and may enable carcinogenesis via epigenetic changes.

Understanding the mechanisms of hypermethylation of genes holds potential for development of biomarkers, and molecules to silence hypermethylation are in development [89,95,96]. The *MSMB* gene, a prostate cell growth regulator noted earlier in this review, may be silenced by EZH2 in androgen-resistant CaP [97]. EZH2 is the functional aspect of a protein complex responsible for catalyzing methylation of histone proteins and histone deacetylase (HDAC-1) [89]. This methylation is one potential mechanism to silence genes controlling prostate differentiation [97]. The *HDAC-1* gene has been noted to be overexpressed in CaP containing the *TMPRSS2-ERG* fusion [98], a common oncogenic activation in CaP (discussed below).

As previously mentioned, epigenetics and the effects on gene expression are likely influenced by environmental and inflammatory changes. Gene expression compared between AA and CA men with CaP has shown significant variation in inflammatory and immune-related genes [99]. Two proteins involved in the inflammatory response, chemokine receptor 4 and matrix metalloproteinase-9 (MMP-9), are more greatly expressed in CaP of AA than European-American men [13]. Evidence also suggests that environmental factors such as diet influence epigenetic processes [100]. Joshi *et al.* [101] recently found that men who have a high intake of red meat and white fish cooked at high temperatures have a significant risk for advanced CaP [101]. Likewise, common intake of deep-fried foods is associated with an increased risk for CaP [102]. In an animal model, mice on an omega-3 fatty acid diet had reduced prostate tumor growth, reduced histological progression, increased apoptosis, and improved survival [103]. The omega-6 fatty acid diet had the opposite, undesired effects on the mice. The authors postulated that diet may have an under-appreciated effect on gene expression [103]. Additionally, CaP cells exposed to serum from mice on a high-fat diet show more invasive features and up-regulated inflammatory proteins, like MMP-9, compared against CaP cells exposed to serum from a lower-fat control diet [104]. Some epidemiologic data do reflect that AA men are more likely to be obese and have a high fat diet than CA men [105], although obesity rates for all ethnicities of Americans are high. This line of research is intriguing, and in the coming years the affects of dietary choices on epigenetics may reiterate the importance of healthy, balanced diet.

## 6. Cancer Genes

Over the past decade, much research has focused on alterations of cancer genes and their effects in CaP [106–108]. Variations in prevalence across ethnicity and race have been noted and continue to improve our understanding of genetic and non-genetic influences on CaP. In the coming years, we may be able to sub-type CaP by causal cancer gene alterations, which will improve our ability to personalize treatment. The following section discusses the most validated CaP gene and its association across race.

### TMPRSS2-ERG Fusions

*TMPRSS2* is an androgen responsive protease specific to prostate cells and ETS Related Gene (ERG) is part of the ETS gene family, which encode transcription factors. *TMPRSS2-ERG* fusion develops exclusively in CaP leading to the overexpression of ERG fusion products, and it is the most common known oncogene in CaP [109,110]. *TMPRSS2-ERG* is commonly cited to be present in 50% of CaP [107]. The fusion may be mediated by (1) the AR in the presence of genotoxic factors or (2) topoisomerase-2b-catalyzed recombination [111,112]. Multiple published reviews discuss *TMPRSS2-ERG* fusion extensively [107,108,113–115]. Since discovery of the *TMPRSS2-ERG* fusion, researchers have attempted to define its role in prostate carcinogenesis, diagnosis, and progression. Overexpression of ERG in a large number of CaP patients has been well described and emerging data have established the causal nature of ERG activation in CaP [107,108]. However, several studies have found conflicting results regarding the effects of *ERG* oncogene when applied to CaP aggressiveness, progression, and outcome [109,116–120]. There is relatively less data on evaluation of ERG oncoprotein in CaP; however, concordance between *ERG* oncogene and oncoprotein has been established [121–123].

Accumulating data suggest that there are differences of *ERG* oncogenic alterations across ethnicities [109,124–126]. Significantly greater *ERG* expression in CA men compared to AA men was noted in initial papers describing *ERG* overexpression and *ERG* splice variants [109,126]. In a study on the utility of a urine-based ERG assay to screen for CaP, the assay performed better for CA compared to AA men, and the performance for CA men improved further when the PSA was ≤ 4 [127]. Magi-Galluzzi *et al.* [124] used tissue microarrays from 42 CA and 64 AA men, and they identified *ERG* fusion by fluorescence in situ hybridization (FISH). In their study, 50% of the CA men and 31% of AA men were positive for *ERG* fusion; however, the predominate type of fusion in CA men was through translocation while AA men most commonly exhibited fusion through transcriptional deletion [124]. These studies suggest that the ERG expression (1) may be significantly more common in CA than AA men, and (2) the mechanism of acquisition may differ across race. These two observations could affect the impact of ERG in the proteome and on the natural history of CaP.

**Figure 1:**
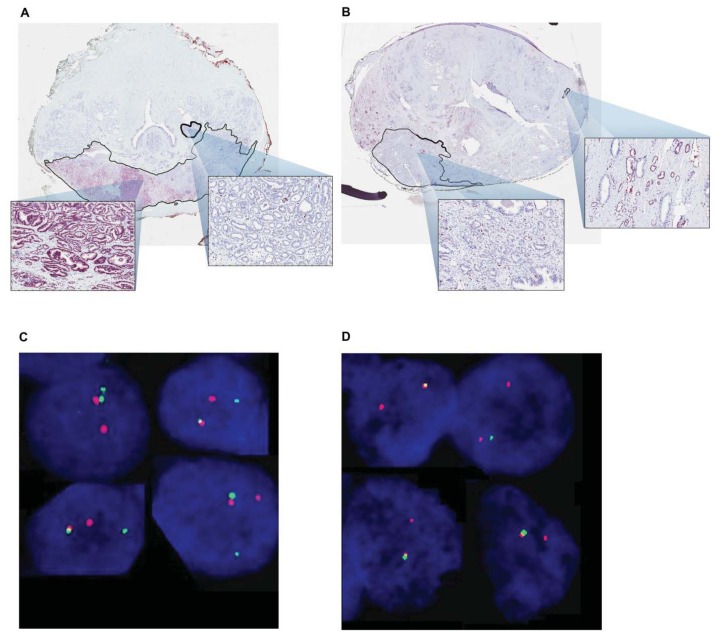
Representative images of whole mount sections analyzed by ERG IHC and FISH in Caucasian American (CA) and African American (AA) patients. (**A**) ERG positive index tumor and ERG negative secondary tumor of a CA patient are outlined, with representative view fields enlarged; (**B**) ERG negative index tumor and secondary ERG positive tumor in AA patient; (**C**) ERG rearrangement by translocation in a CA patient and (**D**) ERG rearrangement by deletion in an AA patient shown by FISH assay (From Rosen *et al.* [125]).

Rosen and colleagues [125] evaluated ERG expression in the proteome by IHC of whole mount prostatectomy specimens. In matched cohorts of 91 CA and 91 AA men, they observed significantly greater prevalence for ERG oncoprotein in CA *vs.* AA patients (66% *vs.* 43%) and in CA index tumors (63% *vs.* 29%). To ensure accuracy of ERG oncoprotein, they demonstrated 99% agreement in 40 FISH and IHC specimens (Figure 1). The difference in oncoprotein expression was significantly more pronounced in higher grade cancers; 59% (10/17) of CA patients were ERG positive while only 10% (2/20) of AA men were ERG positive [125]. In a follow up study, we sought to describe the prevalence of ERG oncoprotein in an appropriately powered, higher grade CaP cohort, stratified by race [128]. Representative whole mount prostate specimens from 63 CA and 63 AA men were evaluated for ERG oncoprotein by IHC. The index tumor in CA men was ERG positive in 31 of 63 patients (49%), which was significantly more common than the 10 of 63 positive (16%) in AA men. CA men were also significantly more likely to have any tumor focus positive for ERG (59% *vs.* 41%) [128].

There is evidence to suggest that there may be interactions between the Vitamin D receptor (VDR), *ERG* oncogene, and ethnicity. Some epidemiologic studies observed that lower vitamin D levels, especially in younger adults, are associated with an increased risk for CaP, and AA men tend to have lower serum vitamin D concentrations than CA men [129,130]. Researchers have demonstrated that VCaP cells (CaP cells that express ERG) exposed to VDR agonists have increased expression of *TMPRSS2-ERG* mRNA. Those cells were associated with reduced VCaP cell growth and reduced *C-MYC* gene expression [131]. In patient cohorts, VDR protein expression and serum vitamin D levels have been measured and applied to clinicopathologic outcomes. Higher VDR expression in CaP was significantly associated with reduced CaP-specific mortality, lower PSA at diagnosis, lower Gleason score and stage, and an increased likelihood of *TMPRSS2-ERG* fusion [132]. These observations provide a potential indirect mechanism which links the increased prevalence of *ERG* fusion in CA men compared to AA men. These emerging data on the ethnic differences of the ERG oncogene are promising in defining biological differences of CaP.

## 7. Conclusions

This review focuses on genetic and molecular differences in CaP between CA and AA men. As discussed previously, the incidence and disease aggressiveness at presentation is worse for AA men than for CA men. While CaP is in all probability affected by sex hormones and environmental influences, genetics also plays an important role in carcinogenesis and progression. Unlocking the molecular differences between races will help us reduce the impact of this disease on AA men and men of all ethnicities. Our ability to understand the carcinogenesis and molecular alterations associated with aggressive disease will help treat CaP more successfully and prevent earlier morbidity and mortality. GWAS studies have been fruitful and have enhanced potential as biomarkers of risk. However, studies to date have not provided risk allele pathways that are better than currently utilized risk models. GWAS have been slowed by the lack of association with known proteins and oncogenic pathways. These pathways are a fairly new, complex and promising field; hopefully, more research will provide insight on gene expression in CaP and racial disparities. Unfortunately, there are very few known molecular mechanisms that show consistent prevalence for either CA or AA men.

In addition, although not discussed in this review, there is a new field of research that provides a landscape of cancer genomes in unprecedented details. Major advances in DNA sequencing technology (“next generation sequencing”) facilitate the entire genome of tumors to be evaluated for genetic abnormalities compared to the normal genomic DNA from the same person [133,134]. This field will likely lead to recognition of novel drivers of cancer mutations and pathways, which may enable targeted individual therapies. The first reports on CaP genome sequencing did not compare AA and CA patients [135–137]. However, the next wave of discoveries regarding genetic and molecular differences in prostate cancer between AA and CA men may very well come from enhanced next generation sequencing studies.

The findings discussed in this review support the potential to stratify CaP by cancer gene alterations. This approach may not only improve our understanding of CaP differences across ethnicities, but also help elucidate the causes CaP and improve its management. Studies that have evaluated the prevalence of ERG across race have shown consistently that ERG is much more common in CA men compared to AA men. Moreover, these differences are described in studies with varied methods of detection and different objectives, which adds weight to the findings.

The search for common cancer genes in AA men continues. As we move toward describing CaP by molecular alterations, use of ERG typing (and other potential cancer genes) for diagnostic and therapeutic roles has the potential to help unravel the biologic differences in CaP across ethnic groups. Since AA men develop CaP most commonly and tend to have more aggressive disease, understanding disease in AA men may unlock important clues to help predict which cancers are aggressive in all ethnicities.

The technology to analyze cancer gene alterations can be utilized in every day clinical practice to sub-type CaP by molecular alterations for individually targeted therapy. Clarification of the prevalence of these alterations across ethnicities, populations, and their impact on CaP must be better defined. This genetic approach will enable clinicians to separate better patients who necessitate treatment from those who can be observed and thus reduce morbidity from overtreatment of this common malignancy.

## References

[1] Siegel R., Naishadham D., Jemal A. (2013). Cancer statistics. CA Cancer J. Clin.

[2] Chornokur G., Dalton K., Borysova M.E., Kumar N.B. (2011). Disparities at presentation, diagnosis, treatment, and survival in African American men affected by prostate cancer. Prostate.

[3] Schwartz K., Powell I.J., Underwood W., George J., Yee C., Banerjee M. (2009). Interplay of race, socioeconomic status, and treatment on survival of patients with prostate cancer. Urology.

[4] Major J.M., Oliver M.N., Doubeni C.A., Hollenbeck A.R., Graubard B.I., Sinha R. (2012). Socioeconomic status, healthcare density, and risk of prostate cancer among African American and Caucasian men in a large prospective study. Cancer Causes Control.

[5] Sridhar G., Masho S.W., Adera T., Ramakrishnan V., Roberts J.D. (2010). Do African American men have lower survival from prostate cancer compared with White men? A meta-analysis. Am. J. Mens. Health.

[6] Cullen J., Brassell S., Chen Y., Porter C., L’Esperance J., Brand T., McLeod D.G. (2011). Racial/ethnic patterns in prostate cancer outcomes in an active surveillance cohort. Prostate Cancer.

[7] Berger A.D., Satagopan J., Lee P., Taneja S.S., Osman I. (2006). Differences in clinicopathologic features of prostate cancer between black and white patients treated in the 1990s and 2000s. Urology.

[8] Kheirandish P., Chinegwundoh F. (2011). Ethnic differences in prostate cancer. Br. J. Cancer.

[9] Odedina F.T., Akinremi T.O., Chinegwundoh F., Roberts R., Yu D., Reams R.R., Freedman M.L., Rivers B., Green B.L., Kumar N. (2009). Prostate cancer disparities in black men of African descent: A comparative literature review of prostate cancer burden among black men in the United States, Caribbean, United Kingdom, and West Africa. Infect. Agents Cancer.

[10] Heath E.I., Kattan M.W., Powell I.J., Sakr W., Brand T.C., Rybicki B.A., Thompson I.M., Aronson W.J., Terris M.K., Kane C.J. (2008). The effect of race/ethnicity on the accuracy of the 2001 Partin Tables for predicting pathologic stage of localized prostate cancer. Urology.

[11] Moul J.W., Sesterhenn I.A., Connelly R.R., Douglas T., Srivastava S., Mostofi F.K., McLeod D.G. (1995). Prostate-specific antigen values at the time of prostate cancer diagnosis in African-American men. JAMA.

[12] Tewari A., Horninger W., Badani K.K., Hasan M., Coon S., Crawford E.D., Gamito E.J., Wei J., Taub D., Montie J. (2005). Racial differences in serum prostate-specific (PSA) doubling time, histopathological variables and long-term PSA recurrence between African-American and white American men undergoing radical prostatectomy for clinically localized prostate cancer. BJU Int.

[13] Wallace T.A., Prueitt R.L., Yi M., Howe T.M., Gillespie J.W., Yfantis H.G., Stephens R.M., Caporaso N.E., Loffredo C.A., Ambs S. (2008). Tumor immunobiological differences in prostate cancer between African-American and European-American men. Cancer Res.

[14] Eichholz A., Ferraldeschi R., Attard G., de Bono J.S. (2012). Putting the brakes on continued androgen receptor signaling in castration-resistant prostate cancer. Mol. Cell. Endocrinol.

[15] Ryan C.J., Tindall D.J. (2011). Androgen receptor rediscovered: The new biology and targeting the androgen receptor therapeutically. J. Clin. Oncol.

[16] Lamont K.R., Tindall D.J. (2011). Minireview: Alternative activation pathways for the androgen receptor in prostate cancer. Mol. Endocrinol.

[17] Ghanadian R., Puah C.M., O’Donoghue E.P. (1979). Serum testosterone and dihydrotestosterone in carcinoma of the prostate. Br. J. Cancer.

[18] Noble R.L. (1977). The development of prostatic adenocarcinoma in Nb rats following prolonger sex hormone administration. Cancer Res.

[19] Bosland M.C., Ford H., Horton L. (1995). Induction at high incidence of ductal prostate adenocarcinomas in NBL/Cr and Sprague-Dawley Hsd:SD rats treated with a combination of testosterone and estradiol-17 beta or diethylstilbestrol. Carcinogenesis.

[20] Ho S.M., Lee M.T., Lam H.M., Leung Y.K. (2011). Estrogens and prostate cancer: Etiology, mediators, prevention, and management. Endocrinol. Metab. Clin. North Am.

[21] Gann P.H., Hennekens C.H., Ma J., Longcope C., Stampfer M.J. (1996). Prospective study of sex hormone levels and risk of prostate cancer. J. Natl. Cancer Inst.

[22] Roddam A.W., Allen N.E., Appleby P., Key T.J. (2008). Endogenous sex hormones and prostate cancer: A collaborative analysis of 18 prospective studies. J. Natl. Cancer Inst.

[23] Bosland M.C., Mahmoud A.M. (2011). Hormones and prostate carcinogensis: Androgens and estrogens. J. Carcinog..

[24] Massengill J.C., Sun L., Moul J.W., Wu H., McLeod D.G., Amling C., Lance R., Foley J., Sexton W., Kusuda L. (2003). Pretreatment total testosterone level predicts pathological stage in patients with localized prostate cancer treated with radical prostatectomy. J. Urol.

[25] Schatzl G., Madersbacher S., Haitel A., Gsur A., Preyer M., Haidinger G., Gassner C., Ochsner M., Marberger M. (2003). Associations of serum testosterone with microvessel density, androgen receptor density and androgen receptor gene polymorphism in prostate cancer. J. Urol.

[26] Yao S., Till C., Kristal A.R., Goodman P.J., Hsing A.W., Tangen C.M., Platz E.A., Stanczyk F.Z., Reichardt J.K., Tang L. (2011). Serum estrogen levels and prostate cancer risk in the prostate cancer prevention trial: A nested case-control study. Cancer Causes Control.

[27] Andriole G.L., Bostwick D.G., Brawley O.W., Gomella L.G., Marberger M., Montorsi F., Pettaway C.A., Tammela T.L., Teloken C., Tindall D.J. (2010). Effect of dutasteride on the risk of prostate cancer. N. Engl. J. Med.

[28] Thompson I.M., Goodman P.J., Tangen C.M., Lucia M.S., Miller G.J., Ford L.G., Lieber M.M., Cespedes R.D., Atkins J.N., Lippman S.M. (2003). The influence of finasteride on the development of prostate cancer. N. Engl. J. Med.

[29] Ellem S.J., Risbridger G.P. (2010). Aromatase and regulating the estrogen:androgen ratio in the prostate gland. J. Steroid Biochem. Mol. Biol.

[30] Price D., Stein B., Sieber P., Tutrone R., Bailen J., Goluboff E., Burzon D., Bostwick D., Steiner M. (2006). Toremifene for the prevention of prostate cancer in men with high grade prostatic intraepithelial neoplasia: Results of a double-blind, placebo controlled, phase IIB clinical trial. J. Urol.

[31] Taneja S.S., Morton R., Barnette G., Sieber P., Hancock M.L., Steiner M. (2013). Prostate cancer diagnosis among men with isolated high-grade intraepithelial neoplasia enrolled onto a 3-year prospective phase III clinical trial of oral toramifene. J. Clin. Oncol.

[32] Ross R., Bernstein L., Judd H., Hanisch R., Pike M., Henderson B. (1986). Serum testosterone levels in healthy young black and white men. J. Natl. Cancer Inst.

[33] Winters S.J., Brufsky A., Weissfeld J., Trump D.L., Dyky M.A., Hadeed V. (2001). Testosterone, sex hormone-binding globulin, and body composition in young adult African American and Caucasian men. Metabolism.

[34] Rohrmann S., Nelson W.G., Rifai N., Brown T.R., Dobs A., Kanarek N., Yager J.D., Platz E.A. (2007). Serum estrogen, but not testosterone levels differ between black and white men in a nationally representative sample of Americans. J. Clin. Endocrinol. Metab.

[35] Orwoll E.S., Nielson C.M., Labrie F., Barrett-Connor E., Cauley J.A., Cummings S.R., Ensrud K., Karlsson M., Lau E., Leung P.C. (2010). Evidence for geographical and racial variation in serum sex levels in older men. J. Clin. Endocrinol. Metab.

[36] Kubricht W.S., Williams B.J., Whately T., Pinckard P., Eastham J.A. (1999). Serum testosterone levels in African-American and white men undergoing prostate biopsy. Urology.

[37] Marks L.S., Hess D.L., Dorey F.J., Macairan M.L. (2006). Prostate tissue testosterone and dihydrotestosterone in African-American and white men. Urology.

[38] Kazemi-Esfarjani P., Trifiro M.A., Pinsky L. (1995). Evidence for a repressive function of the long polyglutamine tract in the human androgen receptor: Possible pathogenetic relevance for the (CAG)n-expanded neuronopathies. Hum. Mol. Genet.

[39] Edwards A., Hammond H.A., Jin L., Caskey C.T., Chakraborty R. (1992). Genetic variation at five trimeric and tetrameric tandem repeat loci in human population groups. Genomics.

[40] Chamberlain N.L., Driver E.D., Miesfeld R.L. (1994). The length and location of CAG trinucleotide repeats in the androgen receptor *N*-terminal domain affect transactivation function. Nucleic Acids Res.

[41] Beilin J., Ball E.M., Favaloro J.M., Zajac J.D. (2000). Effect of the androgen receptor CAG repeat polymorphism on transcriptional activity: Specificity in prostate and non-prostate cell lines. J. Mol. Endocrinol.

[42] Bennett C.L., Price D.K., Kim S., Lui D., Jovanovic B.D., Nathan D., Johnson M.E., Montgomery J.S., Cude K., Brockbank J.C. (2002). Racial variation in CAG repeat lengths within the androgen receptor gene among prostate cancer patients of lower socioeconomic status. J. Clin. Oncol.

[43] Powell I.J., Land S.J., Dey J., Heilbrun L.K., Hughes M.R., Sakr W., Everson R.B. (2005). The impact of CAG repeats in exon 1 of the androgen receptor on disease progression after prostatectomy. Cancer.

[44] Freedman S.J., Isaacs W.B. (2005). Explaining racial differences in prostate cancer in the United States: Sociology or biology?. Prostate.

[45] Sartor O., Zheng Q., Eastham J.A. (1999). Androgen receptor gene CAG repeat length varies in a race-specific fashion in men without prostate cancer. Urology.

[46] Giovannucci E., Stampfer M.J., Krithivas K., Bown M., Dahl D., Brufsky A., Talcott J., Hennekens C.H., Kantoff P.W. (1997). The CAG repeat within the androgen receptor gene and its relationship to prostate cancer. Proc. Natl. Acad. Sci. USA.

[47] Ingles S.A., Ross R.K., Yu M.C., Irvine R.A., La Pera G., Haile R.W., Coetzee G.A. (1997). Association of prostate cancer risk with genetic polymorphisms in vitamin D receptor and androgen receptor. J. Natl. Cancer Inst.

[48] Giovannucci E. (2002). Is the androgen receptor CAG repeat length significant for prostate cancer?. Cancer Epidemiol. Biomarkers Prev.

[49] Freedman M.L., Pearce C.L., Penney K.L., Hirschhorn J.N., Kolonel L.N., Henderson B.E., Altshuler D. (2005). Systematic evaluation of genetic variation at the androgen receptor locus and risk of prostate cancer in a multiethnic cohort study. Am. J. Hum. Genet.

[50] Price D.K., Chau C.H., Till C., Goodman P.J., Baum C.E., Ockers S.B., English B.C., Minasian L., Parnes H.L., Hsing A.W. (2010). Androgen receptor CAG repeat length and association with prostate cancer risk: Results from the prostate cancer prevention trial. J. Urol..

[51] Gilligan T., Manola J., Sartor O., Weinrich S.P., Moul J.W., Kantoff P.W. (2004). Absence of a correlation of androgen receptor gene CAG repeat length and prostate cancer risk in an African-American population. Clin. Prostate Cancer.

[52] Lange E.M., Sarma A.V., Ray A., Wang Y., Ho L., Anderson S.A., Cunningham J.M., Cooney K.A. (2008). The androgen receptor CAG and GGN repeat polymorphisms and prostate cancer susceptibility in African-American men: Results from the Flint Men’s Health Study. J. Hum. Genet.

[53] Foradori C.D., Weiser M.J., Handa R.J. (2008). Non-genomic actions of androgens. Front. Neuroendocrinol.

[54] Gaston K.E., Desok K., Singh S., Ford O.H., Mohler J.L. (2003). Racial differences in androgen receptor protein expression in men with clinically localized prostate cancer. J. Urol.

[55] Sterbis J.R., Gao C., Furusato B., Chen Y., Shaheduzzaman S., Ravindranath L., Osborn D.J., Rosner I.L., Dobi A., McLeod D.G. (2008). Higher expression of the androgen-regulared gene *PSA/HK3* mRNA in prostate cancer tissues predicts biochemical recurrence-free survival. Clin. Cancer Res.

[56] Dobi A., Furusato B., Shaheduzzaman S., Chen Y., Vahey M., Nydam T., Sesterhenn I.A., McLeod D.G., Petrovics G., Srivastava S. (2010). *ERG* expression levels in prostate tumors reflect functional status of the androgen receptor (AR) as a consequence of fusion of *ERG* with AR regulated gene promoters. Open Cancer J.

[57] Kim H.S., Moreira D.M., Jayachandran J., Gerber L., Banez L.L., Vollmer R.T., Lark A.L., Donovan M.J., Powell D., Khan F.M. (2011). Prostate biopsies from black men express higher levels of aggressive disease biomarkers than prostate biopsies from white men. Prostate Cancer Prostatic Dis.

[58] Choudury A.D., Eeles R., Freedland S.J., Issacs W.B., Pomerantz M.M., Schalken J.A., Tammela T.L., Visakorpi T. (2012). The role of genetic markers in the management of prostate cancer. Eur. Urol.

[59] Goh C.L., Saunders E.J., Leongamornlert D.A., Tymrakiewicz M., Thomas K., Selvadurai E.D., Woode-Amissah R., Dadaev T., Mahmud N., Castro E. (2013). Clinical implications of family history of prostate cancer and genetic risk single nucleotide polymorphism (SNP) profiles in an active surveillance cohort. BJU Int..

[60] Zeegers M.P., Jellema A., Ostrer H. (2003). Empiric risk of prostate carcinoma for relatives of patients with prostate carcinoma: A meta-analysis. Cancer.

[61] Pomerantz M.M., Freedman M.L. (2010). Genetics of prostate cancer risk. Mt. Sinai J. Med.

[62] Witte J.S. (2009). Prostate cancer genomics: Toward a new understanding. Nat. Rev. Genet.

[63] Sfano K.S., de Marzo A.M. (2012). Prostate cancer and inflammation: The evidence. Histopathology.

[64] Thomas G., Jacobs K.B., Yeager M., Kraft P., Wacholder S., Orr N., Yu K., Chatterjee N., Welch R., Hutchinson A. (2008). Multiple loci identified in a genome-wide association study of prostate cancer. Nat. Genet.

[65] Bensen J.T., Xu Z., Smith G.J., Mohler J.L., Fontham E.T., Taylor J.A. (2013). Genetic polymorphism and prostate cancer aggressiveness: A case-only study of 1,536 GWAS and candidate SNPs in African-Americans and European-Americans. Prostate.

[66] Amundadottir L.T., Sulem P., Gudmundsson J., Helgason A., Baker A., Agnarsson B.A., Sigurdsson A., Benediktsottir K.R., Cazier J.B., Sainz J. (2006). A common variant associated with prostate cancer in European and African populations. Nat. Genet.

[67] Freedman M.L., Haiman C.A., Patterson N., McDonald G.J., Tandon A., Waliszewska A., Penney K., Steen R.G., Ardlie K., John E.M. (2006). Admixture mapping identifies 8q24 as a prostate cancer risk locus in African-American men. Proc. Natl. Acad. Sci. USA.

[68] Haiman C.A., Patterson N., Freedman M.L., Myers S.R., Pike M.C., Waliszewska A., Neubauer J., Tandon A., Schirmer C., McDonald G.J. (2007). Multiple regions within 8q24 independently affect risk for prostate cancer. Nat. Genet.

[69] Zheng S.L., Sun L., Wiklund F., Smith S., Stattin P., Li G., Adami H.O., Hsu F.C., Zhu Y., Balter K. (2008). Cumulative association of five genetic variants with prostate cancer. N. Engl. J. Med.

[70] Zheng S.L., Sun J., Wiklund F., Gao Z., Stattin P., Purcell L.D., Adami H.O., Hsu F.C., Zhu Y., Adolfsson J. (2009). Genetic Variants and family history predict prostate cancer similar to PSA. Clin Cancer Res.

[71] Thompson I.M., Ankerst D.P., Chi C., Lucia M.S., Goodman P.J., Crawley J.J., Parnes H.L., Coltman C.A. (2005). Operating characteristics of prostate-specific antigen in men with an initial PSA level of 3.0 ng/mL or lower. JAMA.

[72] Hughes L., Ahu F., Ross E., Gross L., Uzzo R.G., Chen D.Y., Viterbo R., Rebbeck T.R., Giri V.N. (2012). Assessing the clinical role of genetic markers of early-onset prostate cancer among high-risk men enrolled in prostate cancer early detection. Cancer Epidemiol. Biomarkers Prev.

[73] Pomerantz M.M., Beckwith C.A., Regan M.M., Wyman S.K., Petrovics G., Chen Y., Hawksworth D.J., Schumacher F.R., Mucci L., Penney K.L. (2009). Evaluation of the 8q24 prostate cancer risk locus and MYC expression. Cancer Res.

[74] Troutman S.M., Sissung T.M., Cropp C.D., Venzon D.J., Spencer S.D., Adesunloye B.A., Huang X., Karzai F.H., Price D.K., Figg W.D. (2012). Racial disparities in the association between variants on 8q24 and prostate cancer: A systematic review and meta-analysis. Oncologist.

[75] Wasserman N.F., Aneas I., Nobrega M.A. (2010). An 8q24 gene desert variant associated with prostate cancer risk confers differential *in vivo* activity to a MYC enhancer. Genome Res.

[76] Fromont G., Godet J., Peyret A., Irani J., Celhay O., Rozet F., Cathelineau X., Cussenot O. (2013). 8q24 amplification is associated with Myc expression and prostate cancer progression and is an independent predictor of recurrence after radical prostatectomy. Hum. Pathol..

[77] Barnabas N., Xu L., Savera A., Hou Z., Barrack E.R. (2011). Chromosome 8 markers of metastatic prostate cancer in African American men: Gain of the MIR151 gene and loss of the NKX3-1 gene. Prostate.

[78] Ding J., Huang S., Wu S., Zhao Y., Liang L., Yan M., Ge C., Yao J., Chen T., Wan D. (2010). Gain of miR-151 on chromosome 8q24.3 facilitates tumour cell migration and spreading through downregulating RhoGDIA. Nat. Cell Biol.

[79] Huppi K., Pitt J.J., Wahlberg B.M., Caplen N.J. (2012). The 8q24 gene desert: An oasis of non-coding transcriptional activity. Front. Genet..

[80] Pomerantz M.M., Shrestha Y., Flavin R.J., Regan M.M., Penney K.L., Mucci L.A., Stampfer M.J., Hunter D.J., Chanock S.J., Schafer E.J. (2010). Analysis of the 10q11 cancer risk locu implicates MSMB and NCOA4 in human prostate tumorigenesis. PLoS Genet..

[81] Whitaker H.C., Kote-Jarai Z., Ross-Adams H., Warren A.Y., Burge J., George A., Bancroft E., Jhavar S., Leongamornlert D., Tymrakiewicz M. (2010). The rs10993994 risk allele for prostate cancer results in clinically relevant changes in microseminoprotein-beta expression in tissue and urine. PLoS One.

[82] Haiman C.A., Chen G.K., Blot W.J., Strom S.S., Berndt S.I., Kittles R.A., Rybicki B.A., Isaacs W., Ingles S.A., Stanford J.L. (2011). Genome-wide association study of prostate cancer in men of African ancestry identifies a susceptibility locus at 17q21. Nat. Genet.

[83] Zeigler-Johnson C.M., Spangler E., Jalloh M., Gueye M., Rennert H., Rebbeck T.R. (2008). Genetic susceptibility to prostate cancer in men of African descent: Implications for global disparities in incidence and outcomes. Can. J. Urol.

[84] Sarma A.V., Dunn R.L., Lange L., Ray A., Wang Y., Lange E.M., Cooney K.A. (2008). Genetic polymorphisms in *CYP17*, *CYP3A4*, *CYP19A1*, *SRD5A2*, *IGF-1*, and *IGFBP-3* and prostate cancer risk in African-American Men: The Flint Men’s Health Study. Prostate.

[85] Wright J.L., Kwon E.M., Lin D.W., Kolb S., Koopmeiners J.S., Feng Z., Ostrander E.A., Stanford J.L. (2010). CYP17 polymorphisms and prostate cancer outcomes. Prostate.

[86] Taioli E., Sears V., Watson A., Flores-Obando R.E., Jackson M.D., Ukoli F.A., de Syllos Colus I.M., Fernandez P., McFarlance-Anderson N., Ostrander E.A. (2013). Polymorphisms in CYP17 and CYP3A4 and prostate cancer in men of African descent. Prostate.

[87] Whitman E.J., Pomerantz M., Chen Y., Chamberlin M.M., Furusato B., Gao C., Ali A., Ravindranath L., Dobi A., Sesterhenn I.A. (2010). Prostate cancer risk allele specific for African descent associates with pathologic stage at prostatectomy. Cancer Epidemiol. Biomarkers Prev.

[88] Bao B.Y., Pao J.B., Huang C.N., Pu Y.S., Chang T.Y., Lan Y.H., Lu T.L., Lee H.Z., Chen L.M., Ting W.C. (2012). Significant associations of prostate cancer susceptibility variants with survival in patients treated with androgen-deprivation therapy. Int. J. Cancer.

[89] Cooper C.S., Foster C.S. (2009). Concepts of epigenetics in prostate cancer development. Br. J. Cancer.

[90] Jeronimo C., Bastian P.J., Bjartell A., Carbone G.M., Catto J.W., Clark S.J., Henrique R., Nelson W.G., Shariat S.F. (2011). Epigenetics in prostate cancer: Biologic and relevance. Eur. Urol.

[91] Nelson W.G., Yegnasubramanian S., Agoston A.T., Bastian P.J., Lee B.H., Nakayama M., de Marzo A.M. (2007). Abnormal DNA methylation, epigenetics, and prostate cancer. Front. Biosci.

[92] Kwabi-Addo B., Chung W., Shen L., Ittman M., Wheeler T., Jelinek J., Issa J.P. (2007). Age-related DNA methylation changes in normal human prostate tissues. Clin. Cancer Res.

[93] Nakayama M., Bennett C.J., Hicks J.L., Epstein J.I., Platz E.A., Nelson W.G., de Marzo A.M. (2003). Hypermethylation of the human glutathione *S*-transferase-pi gene (GSTP1) CpG island is present in a subset of proliferative inflammatory atrophy lesions but not in normal or hyperplastic epithelium of the prostate: A detailed study using laser-capture microdissection. Am. J. Pathol.

[94] Kwabi-Addo B., Wang S., Chung W., Jelinek J., Patierno S.R., Wang B., Andrawis R., Lee N.H., Apprey V., Issa J. (2010). Identification of differentially methylated genes in normal prostate tissues from African American and Caucasion Men. Clinc. Cancer Res.

[95] Bastian P.J., Yegnasubramanian S., Palapattu G.S., Rogers C.G., Lin X., de Marzo A.M., Nelson W.G. (2004). Molecular biomarker in prostate cancer: The role of CpG island hypermethylation. Eur. Urol.

[96] Hopkins T.G., Burns P.A., Routledge M.N. (2007). DNA methylation of GSTP1 as biomarker in diagnosis of prostate cancer. Urology.

[97] Beke L., Nuytten M., van Eynde A., Beullens M., Bollen M. (2007). The gene encoding the prostatic tumor suppressor PSP94 is a target for repression by the polycomb group protein EZH2. Oncogene.

[98] Iljin K., Wolf M., Edgren H., Gupta S., Kilpinen S., Skotheim R.I., Peltola M., Smit F., Verhaegh G., Schalken J. (2006). TMPRSS2 fusions with oncogenic ETS factors in prostate cancer involve unbalanced genomic rearrangements and are associated with HDAC1 and epigenetic reprogramming. Cancer Res.

[99] Rose A.E., Satagopan J.M., Oddoux C., Zhou Q., Xu R., Olshen A.B., Yu J.Z., Dash A., Jean-Gilles J., Reuter V. (2010). Copy number and gene expression differences between African American and Caucasian American prostate cancer. J. Transl. Med..

[100] Ross S.A. (2010). Evidence for the relationship between diet and cancer. Exp. Oncol.

[101] Joshi A.D., John E.M., Koo J., Ingles S.A., Stern M.C. (2012). Fish intake, cooking practices, and risk of prostate cancer: Results from a multi-ethnic case-control study. Cancer Causes Control.

[102] Stott-Miller M., Neuhouser M.L., Stanford J.L. (2013). Consumption of deep-fried foods and risk of prostate cancer. Prostate.

[103] Berguin I.M., Min Y., Wu R., Wu J., Perry D., Cline J.M., Thomas M.J., Thornburg T., Kulik G., Smith A. (2007). Modulation of prostate cancer genetic risk by omega-3 and omega-6 fatty acids. J. Clin. Invest.

[104] Price R.S., Cavazos D.A., de Angel R.E., Hursting D.A., deGraffenried L.A. (2012). Obesity-related systemtic factors promote an invasive phenotype in prostate cancer cells. Prostate Cancer Prostatic Dis.

[105] Liu J., Hickson D.A., Musani S.K., Talegawkar S.A., Carithers T.C., Tucker K.L., Fox C.S., Taylor H.A. (2013). Dietary patterns, abdominal visceral adipose tissue, and cardiometabolic risk factors in African Americans: The Jackson heart study. Obesity.

[106] Prensner J.R., Rubin M.A., Wei J.T., Chinnaiyan A.M., Beyond P.S.A. (2012). The next generation of prostate cancer biomarkers. Sci. Transl. Med..

[107] Rubin M.A., Maher C.A., Chinnaiyan A.M. (2011). Common gene rearrangements in prostate cancer. J. Clin. Oncol.

[108] Sreenath T.L., Dobi A., Petrovics G., Srivastava S. (2011). Oncogenic activation of ERG: A predominant mechanism in prostate cancer. J. Carcinog.

[109] Petrovics G., Liu A., Shaheduzzaman S., Furasato B., Sun C., Chen Y., Nau M., Ravindranath L., Chen Y., Dobi A. (2005). Frequent overexpression of ETS-related gene-1 (*ERG1*) in prostate cancer transcriptome. Oncogene.

[110] Tomlins S.A., Rhodes D.R., Perner S., Dhanasekaran S.M., Mehra R., Sun X.W., Varambally S., Cao X., Tchinda J., Kuefer R. (2005). Recurrent fusion of TMPRSS2 and ETS transcription factor genes in prostate cancer. Science.

[111] Lin C., Yang L., Tanasa B., Hutt K., Ju B.G., Ohgi K., Zhang J., Rose D.W., Fu X.D., Glass C.K. (2009). Nuclear receptor-induced chromosomal proximity and DNA breaks underlie specific translocations in cancer. Cell.

[112] Haffner M.C., Aryee M.J., Toubaji A., Esopi D.M., Albadine R., Gurel B., Isaacs W.B., Bova G.S., Liu W., Xu J. (2010). Androgen-induced TOP2B-mediated double-strand breaks and prostate cancer gene rearrangements. Nat. Genet.

[113] Tomlins S.A., Bjartell A., Chinnaiyan A.M., Jenster G., Nam R.K., Rubin M.A., Schalken J.A. (2009). ETS gene fusions in prostate cancer: From discovery to daily clinical practice. Eur. Urol.

[114] St. John J., Powell K., Conley-LaComb M.K., Chinni S.R. (2012). *TMPRSS2-ERG* fusion gene expression in prostate tumor cells and its clinical and biological significance in prostate cancer progression. J. Cancer Sci. Ther.

[115] Dobi A., Sreenath T., Srivastava S., Wang Z. (2013). Androgen Dependent Oncogenic Activation of ETS Transcription Factors by Recurrent Gene Fusions in Prostate Cancer. Biological and Clinical Implications. Androgen-Responsive Genes in Prostate Cancer.

[116] Nam R.K., Sugar L., Yang W., Srivastava S., Klotz L.H., Yang L.-Y., Stanimirovic A., Encioiu E., Neill M., Loblaw D.A. (2007). Expression of the TMPRSS2:ERG fusion gene predicts cancer recurrence after surgery for localized prostate cancer. Br. J. Cancer.

[117] Attard G., Clark J., Ambroisine L., Fisher G., Kovacs G., Flohr P., Berney D., Foster C.S., Fletcher A., Gerald W.L. (2008). Duplication of the fusion of TMPRSS2 to ERG sequences identifies fatal human prostate cancer. Oncogene.

[118] Darnel A.D., Lafarque C.J., Vollmer R.T., Corcos J., Bismar T.A. (2009). TMPRSS2-ERG fusion is frequently observed in Gleason pattern 3 prostate cancer in a Canadian cohort. Cancer Biol. Ther.

[119] Rajput A.B., Miller M.A., de Luca A., Boyd N., Leung S., Hurtado-Coll A., Fazli L., Jones E.C., Palmer J.B., Gleave M.E. (2007). Frequency of the *TMPRSS2:ERG* fusion is increased in moderate to poorly differentiated prostate cancers. J. Clin. Pathol.

[120] Pettersson A., Graff R.E., Bauer S.R., Pitt M.J., Lis R.T., Stack E.C., Martin N.E., Kunz L., Penney K.L., Ligon A. (2012). The TMPRSS2:ERG rearrangement, ERG expression, and prostate cancer outcomes: A cohort study and meta-analysis. Cancer Epidemiol. Biomarkers Prev.

[121] Park K., Tomlins S.A., Mudaliar K.M., Chiu Y.L., Esgueva R., Mehra R., Suleman K., Varambally S., Brenner J.C., MacDonald T. (2010). Antibody-based detection of ERG rearrangement-positive prostate cancer. Neoplasia.

[122] Furusato B., Tan S.H., Young D., Dobi A., Sun C., Mohamed A.A., Thangapazham R., Chen Y., McMaster G., Sreenath T. (2010). ERG oncoprotein expression in prostate cancer: Clonal progression of ERG-positive tumor cells and potential for ERG-based stratification. Prostate Cancer Prostatic Dis.

[123] Braun M., Goltz D., Shaikhibrahim Z., Vogel W., Bohm D., Scheble V., Sotlar K., Fend F., Tan S.H., Dobi A. (2012). ERG protein expression and genomic rearrangement status in primary and metastatic prostate cancer—A comparative study of two monoclonal antibodies. Prostate Cancer Prostatic Dis.

[124] Magi-Galluzzi C., Tsusuki T., Elson P., Simmerman K., LaFarque C., Esqueva R., Klein E., Rubin M.A., Zhou M. (2011). TMPRSS2-ERG gene fusion prevalence and class are significantly different in prostate cancer of Caucasian, African-American and Japanese patients. Prostate.

[125] Rosen P., Pfister D., Young D., Petrovics G., Chen Y., Cullen J., Bohm D., Perner S., Dobi A., McLeod D.G. (2012). Differences in frequency of ERG oncoprotein expression between index tumors of Caucasian and African American patients with prostate cancer. Urology.

[126] Hu Y., Dobi A., Sreenath T., Cook C., Tadase A.Y., Ravindranath L., Cullen J., Furusato B., Chen Y., Thanqapazham R.L. (2008). Delineation of TMPRSS2-ERG splice variants in prostate cancer. Clin. Cancer Res.

[127] Rice K.R., Chen Y., Ali A., Whitman E.J., Blase A., Ibrahim M., Elsamanoudi S., Brassell S., Furusato B., Stingle N. (2010). Evaluation of the ETS-related gene mRNA in urine for the detection of prostate cancer. Clin. Cancer Res.

[128] Farrell J., Young D., Chen Y., Cullen J., Petrovics G., McLeod D.G., Sesterhenn I.A., Srivastava S. The Prevalence of ERG Oncoprotein Expression in High Grade Prostate Cancer in African American and Caucasian American Patients.

[129] Koh C.M., Bieberich C.J., Dang C.V., Nelson W.G., Yegnasubramanian S., de Marzo A.M. (2010). MYC and prostate cancer. Genes Cancer.

[130] Grant W.B., Peiris A.N. (2012). Differences in vitamin D status may account for unexplained disparities in cancer survival rates between African and white Americans. Dermatoendocrinology.

[131] Washington M.N., Weigel N.L. (2010). 1α,25-Dihydroxyvitamin D3 inhibits growth of VCaP prostate cancer cells despite inducing the growth-promoting TMPRSS2:ERG gene fusion. Endocrinology.

[132] Hendrickson W.K., Flavin R., Kasperzyk J.L., Fiorentino M., Fang F., Lis R., Fiore C., Penney K.L., Ma J., Kantoff P.W. (2011). Vitamin D receptor protein expression in tumor tissue and prostate cancer progression. J. Clin. Oncol.

[133] Vogelstein B., Papadopoulos N., Velculescu V.E., Zhou S., Diaz L.A., Kinzler K.W. (2013). Cancer genome landscapes. Science.

[134] Garraway L.A., Lander E.S. (2013). Lessons from the cancer genome. Cell.

[135] Berger M.F., Lawrence M.S., Demichelis F., Drier Y., Cibulskis K., Sivachenko A.Y., Sboner A., Esgueva R., Pflueger D., Sougnez C. (2011). The genomic complexity of primary human prostate cancer. Nature.

[136] Barbieri C.E., Demichelis F., Rubin M.A. (2012). Molecular genetics of prostate cancer: Emerging appreciation of genetic complexity. Histopathology.

[137] Grasso C.S., Wu Y.M., Robinson D.R., Cao X., Dhanasekaran S.M., Khan A.P., Quist M.J., Jing X., Lonigro R.J., Brenner J.C. (2012). The mutational landscape of lethal castration-resistant prostate cancer. Nature.

